# Post coital aortic dissection: a case report

**DOI:** 10.1186/1752-1947-2-6

**Published:** 2008-01-16

**Authors:** Gareth Morris-Stiff, Mari Coxon, Elizabeth Ball, Michael H Lewis

**Affiliations:** 1 Department of Surgery, Royal Glamorgan Hospital, Ynysmaerdy, Llantrisant, Wales, UK

## Abstract

**Background:**

Sudden onset peri- or post-coital cardiovascular disease is well documented in the literature including myocardial infarction, pulmonary embolus and subarachnoid haemorrhage. The occurrence of aortic dissection in this setting has been reported only once previously.

**Case presentation:**

We report the case of a 47 year old man who developed sudden onset right leg pain during coitus. This was initially believed to be neurological due to nerve impingement but an MRI failed to identify a prolapse. On further review after 6 weeks, pulses were noted to be absent in the patient's right leg and an urgent vascular review with investigation identified a dissection of the aorta which was subsequently successfully treated.

**Conclusion:**

This case illustrates a rare presentation of aortic dissection and demonstrates the importance of a thorough vascular assessment in the presence of sudden onset limb pain.

## Introduction

Aortic dissection is characterised by separation of the layers of the aortic wall by extraluminal blood that enters the aortic wall, almost invariably through a luminal tear [1]. Despite a reduction in its incidence as a result of improved pharmacological control of hypertension, when it presents acutely, aortic dissection usually has a catastrophic outcome. This case reports an unusual mode of presentation and illustrates the multidisciplinary aspects of the pre-, peri- and post-operative care of an unusual presentation of the condition.

## Case presentation

A 47 year old gentleman presented to his general practitioner with acute onset lower back pain. The pain had commenced during coitus and radiated down the right leg. The initial diagnosis was of acute disc prolapse and he was referred for an urgent neurosurgical opinion. The neurosurgeon concurred that the pain may well have been of neurological origin and arranged an MRI scan. This was reported as showing no evidence of spinal cord pathology. The patient was reassured with the results of the MRI findings and was advised the pain was probably musculoskeletal in origin and should settle. Over the subsequent 6 weeks, the pain persisted and indeed increased in severity. The patient noted claudication-type pain in his right leg after approximately 100 metres. As the pain had not resolved after 6 weeks he revisited his general practitioner. During the subsequent examination the pulses in his right leg were noted to be absent and he was referred for an urgent vascular surgical opinion.

**Figure 1:**
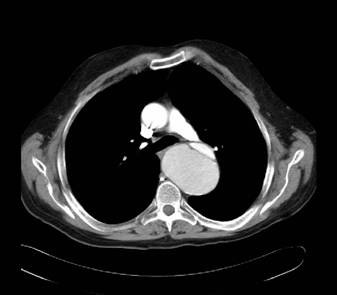
CT scans demonstrating the dissection at the level of the aortic valve.

**Figure 2:**
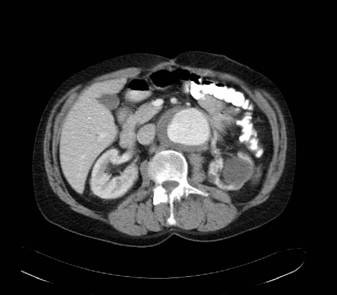
CT scans demonstrating the dissection at the level of the coeliac axis.

**Figure 3:**
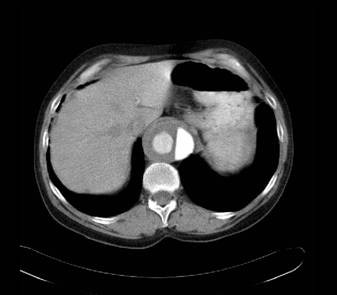
CT scans demonstrating the dissection at the level of the renal arteries.

The patient was seen the following day in the vascular clinic where a history of severe acute claudication-type pain was noted in the right leg. There was a past medical history of marked hypertension and hyperlipidaemia, for which he took relevant medications, but none of angina, myocardial infarct or valvular heart disease. On clinical examination the heart rate was 68 beats per minute regular. The blood pressure in the right arm 130/70 mmHg was lower than that of the left arm 160/80. Cardiac examination was normal. There was no clinical evidence of an abdominal aortic aneurysm. Examination of the limbs revealed that the right lower limb pulses were all absent whilst those of the left leg were present and of good volume. An urgent abdominal ultrasound scan was arranged which demonstrated dissection of the intra-abdominal aorta and a subsequent CT scan (Figures 1, 2, 3) confirmed that the dissection was a Type A dissection extending from the aortic valve down to the aortic bifurcation. A dissection flap was identified in the ascending aorta and also in the postero-inferior aspect of the descending aorta. Both lumens were noted to have flow within them with the true lumen supplying the celiac axis, superior mesenteric artery and right renal artery and the false lumen supplying the left renal artery and inferior mesenteric artery. Immediately below the inferior mesenteric artery the false lumen obliterated.

An immediate opinion was sought at the regional cardiothoracic unit and the patient was transferred urgently under their care. A transthoracic echocardiogram was performed which confirmed the CT findings demonstrating turbulent flow in the ascending aorta suggestive of an intimal tear in the region although the lesion itself was not seen. The arch was mildly dilated but with no visible flap. Flow in the descending aorta was turbulent in the initial 2–3 cm suggesting intimal disruption.

He underwent operative repair of the thoracic dissection on the next available theatre list. The aortic valve was resuspended and the ascending aorta were replaced using an elephant trunk graft with reimplantation of the brachiocephalic artery on 1 patch and the left common carotid and subclavian arteries on a second patch.

After 48 hours in the intensive care unit the patient was transferred to the ward where he made an uneventful recovery. Cardiac and cerebral functions were not impaired by the procedure as evidenced by return to preoperative state and no requirements for chronotropic medications.

A routine postoperative CT scan demonstrated that the repair was satisfactory. There was thrombosis within the false lumen of the descending aorta but persistence of flow within both lumens of the abdominal aorta.

## Discussion

Aortic dissection is characterised by separation of the layers of the aortic wall due to extraluminal blood that has entered the aortic wall through an intimal tear. Tears are seen at areas of high stress, the most common being in the anterior aortic wall just above the aortic valve (66%), and the posterior wall of the proximal descending aorta (33%). When blood enters through an intimal tear it passes longitudinally along the tunica media separating the intima from the adventitia.

An acute dissection of the aorta is one which presents within 14 days of the onset of the disease process. In this case, presentation with new onset back and right leg pain occurred on the first day of symptoms and urgent investigations were instituted.

There are several different formats of classification for thoracic dissection, the most commonly used being that of DeBakey [2], which divides the dissections into 3 types: I – involving the ascending aorta and a variable amount of descending or thoraco-abdominal aorta; II – dissection limited to the ascending aorta; and III – dissection of the descending aorta either without (IIIa) or with (IIIb) involvement of the abdominal aorta. Our patient clearly had a type I dissection with 2 lumina identified running from the aortic valve all the way to the bifurcation of the aorta into common iliac arteries.

The typical presentation of acute dissection is with sudden onset, unexpected, intense pain in the interscapular region radiating to the lower back or abdomen [3]. Patients are typically hypertensive middle-aged or elderly men and the diagnosis should certainly be entertained in patients with such symptoms along with other differential diagnoses which include myocardial ischaemia and abdominal aortic aneurysm. However, as the dissection can affect any of the arteries arising from the aorta, other presentations include stroke, a pulseless limb or abdominal organ dysfunction such as renal failure or intestinal ischaema. In this case, whilst the demographics were typical, the site of the pain was not, being a lot lower than for a typical dissection. Dissection involving the ascending aorta (types I and II) is more hazardous than type III because of the risks of intra-pericardial rupture, acute aortic insufficiency or occlusion of the coronary arteries. For all dissections there is the risk of aortic rupture and ischaemic complications, particularly if the abdominal aorta is affected by the dissection. The patient reported was therefore fortuitous not to have developed complications during the 6 week interval from injury to diagnosis of dissection.

This case has illustrated the importance of taking a detailed history and performing a thorough clinical examination in all patients with acute onset limb pain. In this case, the temporal relationship between the onset of pain and radiation down the right leg would suggest that the dissection into the intra-abdominal portion of the aorta occurred during coitus although it is impossible to prove this. Had the dissection progressed over a period of weeks it is likely that the pain would have progressed gradually rather than arising acutely and persisting at a constant intensity. However, whilst the main injury may have occurred with the initial pain, there may have been slow progression over a period of 6 weeks with loss of the right limb pulses during this period.

The identification of impaired peripheral pulses is another important finding in relation to a diagnosis of aortic dissection. In an extensive examination of the medical literature, Klompas [4] highlighted the importance of obtaining an accurate history noting that 31% of patients had evidence of a pulse deficit, and that for patients with a history highly suggestive of dissection who underwent advanced imaging studies, the positive likelihood ratio of a pulse deficit between contralateral limbs was 5.7 and the negative likelihood ratio was 0.7.

The mode of presentation of our patient's dissection is not classical; however, this is not the first such case. An unfortunate individual reported by Lovas and Silver [5] also ruptured a berry aneurysm during his activities and his outcome was less satisfactory. Furthermore, it would appear from the literature that peri-coital acute vascular disease is not uncommon including myocardial infarction, pulmonary embolism and intracranial haemorrhage. Less surprising is the fact that much of the data has accumulated from the Scandinavian countries.

The pathophysiology of the dissection during coitus is probably related to the well-recognised increases of blood pressure seen during vigorous exercise [6]. It has been demonstrated in a rat model that central aortic pressure increases by up to 19% during exercise [7]. Furthermore, it has been shown in an animal aortic aneurysm model that exercise leads to increased turbulent flow within the aorta and this in turn increases shear pressures on the aortic wall [8].

Once the diagnosis is suspected, the initial management is to initiate full monitoring including heart rate, blood pressure, urine output and central venous pressure. The systolic blood pressure should be reduced to around 100–120 mmHg to prevent further dissection and β-blockade should be instituted. The diagnosis needs to be confirmed by means of a CT scan or trans-oesophageal echocardiography. This provides an assessment of the risk of impending rupture and allows a decision to be made with regards the urgency and type of operation necessary. Options include open replacement of the aorta with reimplantation of arteries with or without valve replacement depending upon the location and extent of the dissection. More recently, the use of endovascular stenting has been documented for aortic dissection. In a proportion of cases, in particular those with a more chronic history and minimal symptoms, conservative management may be adequate.

In our patient there was good flow in both the true and false lumina and so the cardiothoracic surgeons decided not to replace the infra-diaphragmatic aorta. We are therefore left with the dilemma of what to do this segment of aorta – to replace or not?

## Conclusion

We report an unusual mode of presentation of a rare and often fatal condition. This case illustrates the importance of a thorough vascular assessment in the presence of sudden onset limb pain. It also emphasises the importance of multidisciplinary care within surgery vital inputs from cardiologists, radiologists, anaesthetists and intensivists as well as cardiothoracic and vascular surgeons.

## Competing interests

The author(s) declare that they have no competing interests.

## Authors' contributions

The original idea was that of G Morris-Stiff and MH Lewis (consultant in charge of the case). The manuscript was written by M Coxon and E Ball and the manuscript was edited by G Morris-Stiff. The manuscript was approved by all 4 authors prior to submission.

## Consent

The authors confirm that written informed consent was obtained from the patient for publication of the manuscript.
